# The Genesis of the Feeble-Minded

**Published:** 1913-02

**Authors:** 


### Department of Eugenics.

Conducted by Dr. Malone Duggan and Dr. Theo. Y. Hull.

### THE TEXAS STATE SOCIETY OF SOCIAL HYGIENE.

Headquarters: San Antonio, Texas.

DR. MALONE DUGGAN, President, San Antonio.
DR. F. E. DANIEL, 1st Vice President, Austin.
PROF. A. CASWELL ELLIS, 2d Vice President, Austin.
MRS. ELI HERTZBERG, 3d Vice President, San Antonio.
DR. THEO. Y. HULL, Secretary, San Antonio.
MR. W. L. EVANS, Treasurer, San Antonio.

EXECUTIVE COMMITTEE.

Dr. Malone Duggan, Chm.
Dr. Theo. Y. Hull, Sec'y.
Dr. W. F. McCaleb.
Hon. C. H. Jenkins.
Rev. A. W. S. Garden.
Hon. J. C. Willacy.
Mr. W. L. Evans.
Dr. Frank D. Boyd.
Dr. J. R. Nichols.
Dr. J. M. McCutchan.
Dr. W. A. King.
Dr. J. W. Torbett.
Dr. F. C. Walsh.
Dr. Joe Reuss.
Dr. Frank Paschal.

### The Genesis of the Feeble-Minded.

One-half of all feeble-minded persons are born of feeble-minded parents (and there are over 300,000 in institutions in the United States).

Acquired feeble-mindedness is the direct result of racial poisons (alcolene-syphilis, etc.), overwork and overstrain of mothers—"industrial slaves." The offspring issuing from these conditions are nervous. They grow up and become addicted to narcotics to quiet their nerves. Their offspring are neurotic. From these neurotics come the feeble-minded.

